# Effect study of heat treatment on tensile properties of coarse sandstone

**DOI:** 10.1038/s41598-022-21164-w

**Published:** 2022-10-20

**Authors:** Yushun Yang, Sijiang Wei, Jihua Zhang, Jingke Wu, Chunlei Zhang

**Affiliations:** 1 Faculty of Architecture and Civil Engineering, Huaiyin Institute of Technology, Huai’an, 223001 China; 2 School of Energy Science and Engineering, Henan Polytechnic University, Jiaozuo, 454003 China; 3 School of Transportation, Southeast University, Nanjing, 211189 China

**Keywords:** Civil engineering, Natural hazards

## Abstract

Brazilian split experiments were carried out on coarse sandstone, obtained from a coal seam roof passed by the Shihuoshan tunnel in Xinjiang, and treated at different temperatures (room temperature 25 °C and high temperature 100 °C ~ 900 °C). The physical and mechanical characteristics of the samples were studied. The results showed that: after heat treatment, the color of the coarse sandstone samples gradually changed from dark gray to brownish red-pink; the higher the treatment temperature was, the darker the sample color. Microcracks and mineral composition changes occured in the coarse sandstone samples after heat treatment, which decreased the longitudinal wave velocity of the samples. The longitudinal wave velocity of the coarse sandstone samples decreased as a quadratic function of the treatment temperature. With the increase in longitudinal wave velocity, the tensile strength of the samples first increased and then decreased, changing as a quadratic function relationship. After heat treatment, the tensile stress–strain curve of the coarse sandstone could be divided into compaction, elasticity, plasticity, and failure stages. The energy was continuously accumulated in the coarse sandstone before its failure, and it was released suddenly after the failure. With increasing treatment temperature, the cumulative energy in the prepeak stages first increased and then decreased, reaching a maximum value at 500 °C. The prepeak energy and tensile strength of the coarse sandstone samples satisfied a linear function fitting relationship, indicating that a higher tensile strength in the coarse sandstone, led to more accumulated energy in the samples.

## Introduction

With the rapid development of underground engineering construction, engineering problems such as boring tunnels through rocks such as sandstone are increasing. Underground fires affects the physical and mechanical properties of surrounding rock materials and causes systemic failures and catastrophic accidents. Therefore, the mechanical behavior of rock at high temperatures is an important parameter^1–4^. The influence of high temperature on the mechanical properties of rock should be considered when designing of surrounding rock supports after underground engineering fires.

In order to assess the mechanical properties of rocks subjected to high temperatures, researchers have performed numerous studies and achieved important results. With increasing temperature, the attached water and ore-bound water in limestone gradually disappears, and the mineral phase transformation, decomposition and chemical bond fracture increase the internal damage and porosity of the limestone^5^. The stress–strain curves form a hysteresis loops^6^. The strength of limestone decreases with increasing temperature, the angle of internal friction increases first and then decreases with increasing temperature, and the relationship between cohesion and temperature was opposite to that of the angle of internal friction^7^. With increasing heat treatment temperature, the mass, density, P-wave velocity and peak strength of granite generally show a downward trend, and with increasing temperature and confining pressure, the deformation of granite changes from brittle to plastic^8–12^. Cracks were formed at the ends of pores and rapidly expand and connect to form a pore fracture network structure, and the number and size of cracks produced by granite increase with increasing temperature^13–16^^.^ After high-temperature treatment, the permeability of granite gradually increases with the experienced temperature, the increase of permeability within 500 °C was small, and the permeability of granite between 500 and 600 °C undergoes a step change^17^. The permeability of granite increases exponentially with the expansion of fractures^18^. However, in practical engineering, high temperature causes a rock mass to break in tension, the stability decreases and causes damage, and the tensile strength of granite generally decreases with increasing temperature^19–21^. Chaudhary et al.^22^ evaluated in detail some physico-mechanical parameters of sandstone rocks from Eastern India, and their statistical correlation and swift prediction. Tmca et al.^23^ studied high-temperature fluid rock interactions recorded in a serpentinized wehrlite from eastern Singhbhum Craton, India, and their results indicated that these rocks experienced fluid rock interactions at temperatures above the serpentinization process.

The damage mechanisms of granite and sandstone in the thermal damage process are obviously different^24^, resulting in a significant difference in the strength characteristics of the two rocks. Su et al.^25,26^ studied the deformation and strength characteristics of coarse sandstone and fine sandstone after high-temperature exposure, analyzed the relationships among sample deformation, strength and failure characteristics, temperature and confining pressure, and determined the mineral formation in the fine sandstone sample after high temperature polymorphism. The above scientists conducted a series of studies and assessments on limestone, granite, sandstone and shale. Even if the rock engineering was subjected to slight stretching after high-temperature exposure, collapse or other forms of failure will occur. Therefore, there was certain practical value in studying the tensile properties of coarse sandstone after high-temperature treatment. In this paper, coarse sandstone from a coal seam roof passed by the Shihuoshan tunnel, in Xinjiang, China was taken as the research object, and the variation law of tensile strength after being subjected to different temperatures was experimentally studied, to provide theoretical support for the stability of geotechnical engineering after fire exposure.

## Materials and methods

### Coarse sandstone samples

Coarse sandstone samples were taken from a coal seam roof passed by the Shihuoshan tunnel in Xinjiang, China. The local area of the tunnel passes through a natural coal seam zone. The engineering design needs to measure the mechanical parameters of the coarse sandstone samples obtained from the coal seam roof in this area after heat treatment. According to the methods recommended by the ISRM, the coarse sandstone was processed into Brazilian disk samples with a diameter of 50 mm and a height of 40 mm. There were 30 samples in total. The particle size of the samples was 0.2 ~ 1.0 mm, and the average particle size was 0.5 mm; the density of the samples was 2275 ~ 2410 kg/cm^3^, and it’s the average value was 2346 kg/cm^3^, and the dispersion coefficient was 1.4%. There were no obvious joints, cracks or other defects on the surface of the samples, and their macroscopic appearance was relatively uniform.

**Figure 1:**
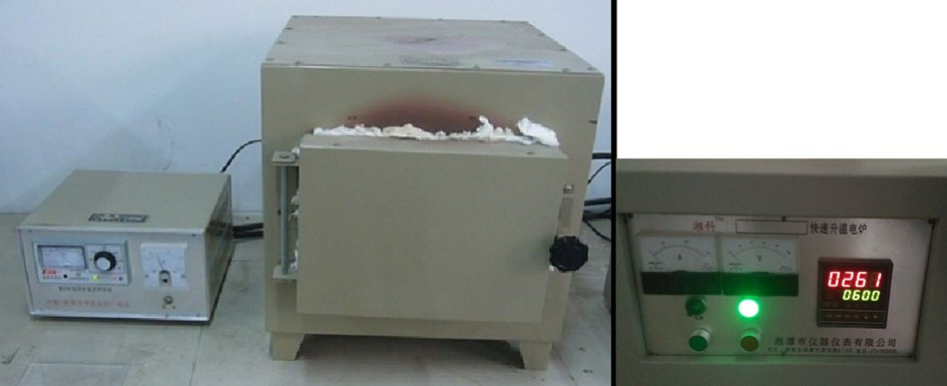
High temperature box resistance furnace.

**Figure 2:**
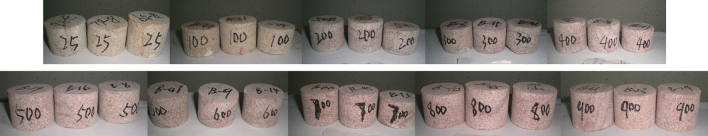
Samples treated at different temperatures.

Samples were treated at different temperatures in a KSW-5D-12 high-heat box-type furnace, as shown in Fig. 1. Except for the temperature of 25 °C, the treatment temperature was set to 100 °C, 200 °C, 300 °C, 400 °C, 500 °C, 600 °C, 700 °C, 800 °C, and 900 °C; each temperature setting uses three samples. To heat the samples evenly, they were slowly heated to the different set temperature at the rate of 10 °C/min, kept at a constant temperature for 4 h, and then cooled to room temperature naturally in the furnace to prepare the coarse sandstone samples treated at different temperatures, as shown in Fig. 2.

### Test methods

*Acoustic wave test* A UTA-2000A acoustic wave tester was used to test the acoustic wave variation mechanism of the coarse sandstone samples treated at different temperatures.

**Figure 3:**
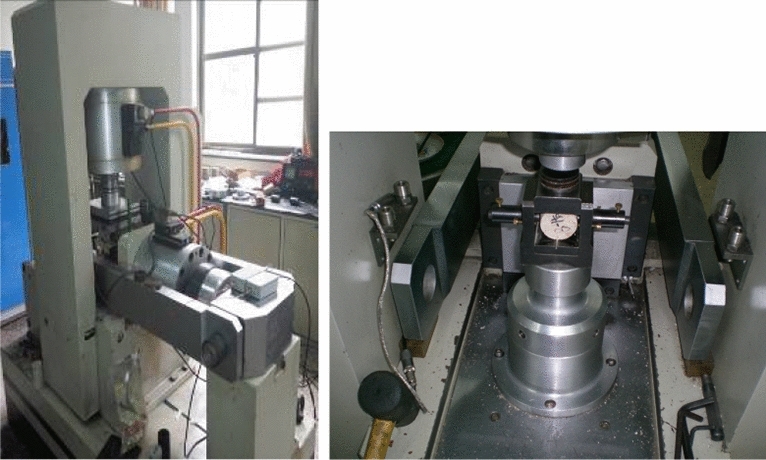
RMT-150B electrohydraulic servo rock test system.

*Brazilian split test* An RMT-150B electrohydraulic servo test system was used, and the test device is shown in Fig. 3. The test device mainly adopts the steel wire split method. The diameter of the steel wire washer was 2 mm, a 5 mm displacement sensor measures the axial displacement of the samples, and a 100 kN force sensor measures the radial load of the samples. The loading process in the test adopts displacement control, and the samples were loaded at a loading rate of 0.002 mm/s until they fail, and three samples were tested at each set temperature.

A D8-ADVNCE X-ray diffractometer was used to analyze and obtain the main mineral components of the coarse sandstone samples treated at different temperatures.

### The Brazilian split mechanism

According to the Boussinesq analytical solution of the concentration force acting on a semi-infinite body in elastic mechanics, a force was applied to both ends of the diameter of the Brazilian disk samples and deducted from the stress generated around the disk samples because of the concentration force. In this way, the horizontal tensile stress at the center point of the disk samples was obtained, which was used as the tensile strength of the samples. Through theoretical derivation, the maximum tensile stress acting on the center of the samples when it fails can be obtained as follows:

1 $$\sigma_{t} = - \frac{2P}{{\pi DL}}$$

where *P* is the load (kN), *D* is the diameter of the disk samples (mm), *L* is the height of the disk samples (mm).

## Experiment results and analyses

### Apparent color variation of samples

Figure 2 shows that the color of the coarse sandstone samples changes significantly after different heat treatments. The color of the samples at room temperature was dark gray. When the treatment temperature was below 500 ℃, with the increase in temperature, the color of the samples gradually changes from dark gray to brownish red. As the temperature increases above 500 ℃, the color of the samples changes to pink; the higher the temperature was, the darker the red color. It was caused by oxidation of the sample due to the high temperature. There were no visible cracks or disintegrations on the samples surfaces, and they were still intact.

### Effect of temperature on the mass change rate of samples

**Figure 4:**
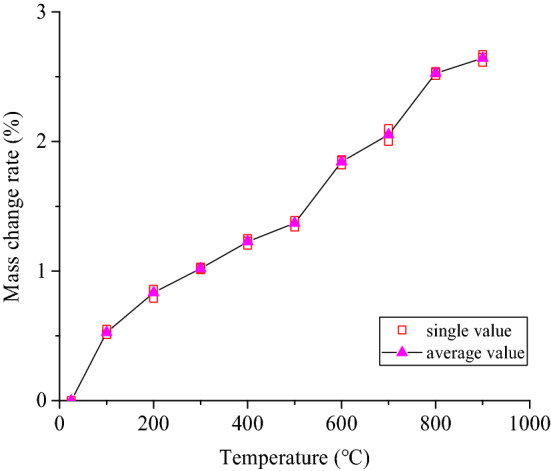
Relationship between the treatment temperatures and the mass change rate of the coarse sandstone samples.

The internal mineral components of the coarse sandstone samples treated at different temperatures were thermally decomposed, and their thermal decomposition degrees differ. The mass change degree of the coarse sandstone samples treated at different temperatures was characterized by the mass change rate (the percentage of the mass reduction of the samples after different high-temperature treatments to the mass of original samples). The relationship between the mass change rate of the samples and the treatment temperature is shown in Fig. 4. The sample mass change rate was positively correlated with the treatment temperature. When the treatment temperature was below 900 °C, the mass change rate of the samples increases gradually with increasing treatment temperature. The average increase in the mass change rate of the samples treated at each set temperature was 0.53%, 0.83%, 1.02%, 1.23%, 1.37%, 1.84%, 2.05%, 2.53%, and 2.64%. With increasing treatment temperature, the water evaporation phenomena in the samples turn into the mineral thermal decomposition and recrystallization in the samples, causing the sample mass to continue to decrease.

### Longitudinal wave velocity

The longitudinal wave velocities of the coarse sandstone samples treated at different temperatures are shown in Table 1.

**Table 1:** Longitudinal wave velocities of the coarse sandstone samples treated at different temperatures.

Temperature	No	25	100	200	300	400	500	600	700	800	900
v/(m/s)	1#	3306	3210	2764	2697	2440	2067	1640	1616	1383	1261
	2#	3293	3245	2801	2740	2470	2222	1641	1646	1442	1316
	3#	3230	3269	3003	2809	2586	2342	1651	1659	1463	1361
	Average value	3276	3241	2856	2749	2499	2210	1644	1640	1429	1313

**Figure 5:**
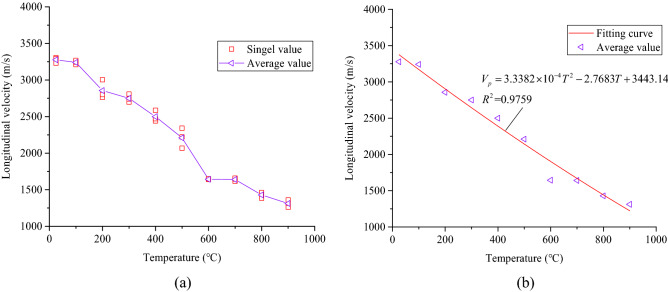
Relationship between the longitudinal wave velocity of coarse sandstone samples and its treatment temperature.

With increasing treatment temperature, the longitudinal wave velocity of the samples decreases slowly. Compared with the samples at 25℃, the longitudinal wave velocities of the samples treated at the different temperatures decreased by 1.08%, 12.84%, 16.11%, 23.74%, 32.54%, 49.83%, 49.94%, 56.38%, and 59.94%, respectively. This indicates that the microstructure and mineral composition of the coarse sandstone samples change. The cementitious substances inside the samples were damaged, and their stiffness was reduced, making the longitudinal wave velocity significantly decrease. The longitudinal wave velocity was a comprehensive macroscopic manifestation of microscopic factors such as rock deformation and failure and the distribution of cracks. The generation of tiny cracks inside the samples reduces its longitudinal wave velocity^24^. The relationship between the longitudinal wave velocity of the samples and the treatment temperature is shown in Fig. 5.

Figure 5a shows that the longitudinal wave velocity of the coarse sandstone samples decreases gradually with incrsasing treatment temperature. When the treatment temperature was 200 °C and 600 °C, the longitudinal wave velocity of the samples reduces significantly. As shown in Fig. 5b, the longitudinal wave velocity of the samples and the treatment temperature satisfy the quadratic function fitting relationship, as shown in Eq. 2.

2 $$V_{p} = 3.3382 \times 10^{ - 4} T^{2} - 2.7683T + 3443.14$$

where *V*_p_ is the longitudinal wave velocity (m/s), *T* is the treatment temperature (°C).

### Damage characteristics

The longitudinal wave velocity of the coarse sandstone samples reflects the damage characteristics of the samples. Therefore, the ratio of the longitudinal wave velocity of the samples at different treatment temperatures to that of the samples at 25 °C was used as the damage variable *D*. In this way, the damage evolution characteristics of the samples were obtained as shown in Eq. (3).

3 $$D(T) = 1 - {{V_{pT} } \mathord{\left/ {\vphantom {{V_{pT} } {V_{p0} }}} \right. \kern-\nulldelimiterspace} {V_{p0} }}$$

where *D*(T) is the damage variable at temperature *T*, *V*_pT_ is the longitudinal wave velocity at temperature *T* (m/s), *V*_p0_ is the longitudinal wave velocity at 25 °C (m/s).

**Figure 6:**
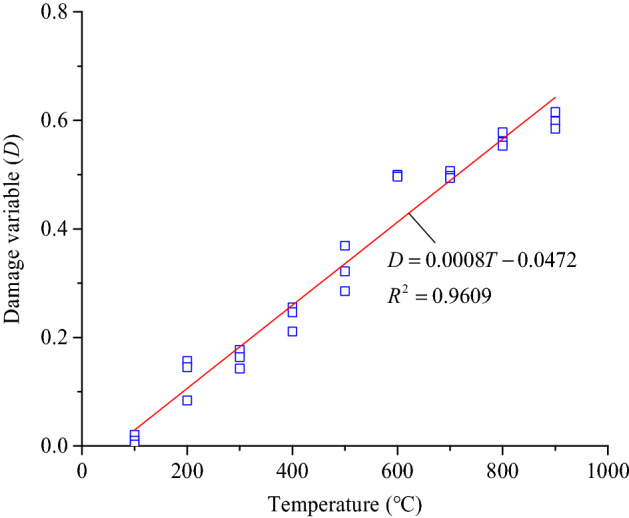
Relationship between the damage variable and the treatment temperature.

According to Eq. 3, the damage variable-treatment temperature relationship curves were drawn, as shown in Fig. 6.

Figure 6 shows that the relationship between the damage variables of the samples treated at different temperatures and the treatment temperature satisfies a linear function as shown in Eq. 4. The correlation coefficient was more than 96%, and the fitting effect was excellent.

4 $$D = 0.0008T - 0.0472.$$

### Deformation characteristics

**Figure 7:**
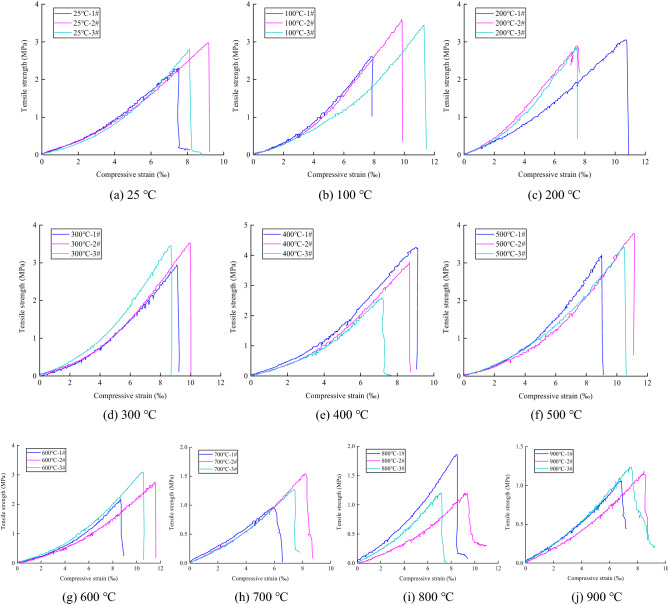
The tensile stress–compressive strain curves of the coarse sandstone samples treated at different temperatures.

The tensile stress–compressive strain curves of the coarse sandstone samples treated at different temperatures are shown in Fig. 7. The abscissa was the ratio of the total compressive deformation value in the loading direction to the diameter of the samples (average compressive strain).

The tensile stress–compressive strain curve was roughly divided into the following stages: compaction, elasticity, plasticity, and failure. Compaction stage: The tensile stress–compressive strain curve was concave upward, and the micropores inside the samples were compacted and closed under the load. Elastic stage: The tensile stress–compressive strain curve approximates a straight line, showing the elastic properties of coarse sandstone. Plastic stage: As the load increases, the coarse sandstone’s interior gradually breaks down, and the tensile stress–compressive strain curve was concave. Failure stage: The load reaches the ultimate capacity of the coarse sandstone, forming a crack that runs through the center of the disc samples. The coarse sandstone loses its bearing capacity due to its instability and failure, and the stress drops rapidly.

**Figure 8:**
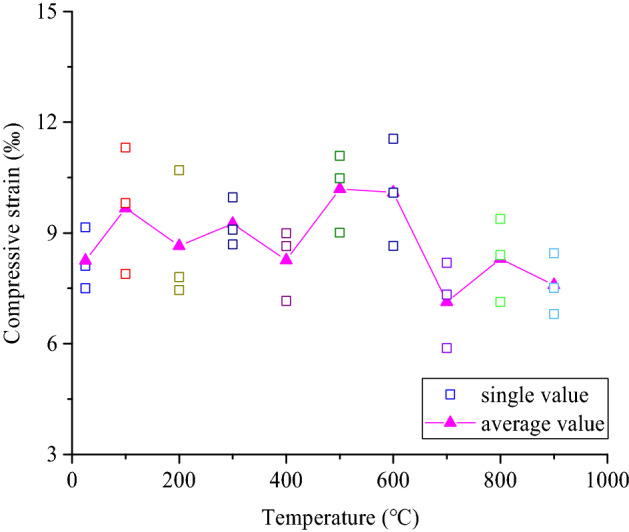
Relationship between the peak compressive strain and the treatment temperature of coarse sandstone samples.

Figure 8 presents the relationship between peak compressive strain and temperature of coarse sandstone sample. With increasing temperature, the peak compressive strain of the coarse sandstone samples fluctuates, but shows a decrease as a whole. Compared with the normal temperature at 25 ℃, the peak compressive strain change rate of coarse sandstone at each temperature was 17.17%, 4.81%, 12.04%, 0.13%, 23.51%, 22.34%, − 13.57%, 0.61%, and -8.07% (the "-" means decrease). Due to the anisotropy of coarse sandstone samples, the internal damage characteristics of samples after high temperature were different, which makes the tensile strength was different, and its deformation characteristics also change accordingly.

**Figure 9:**
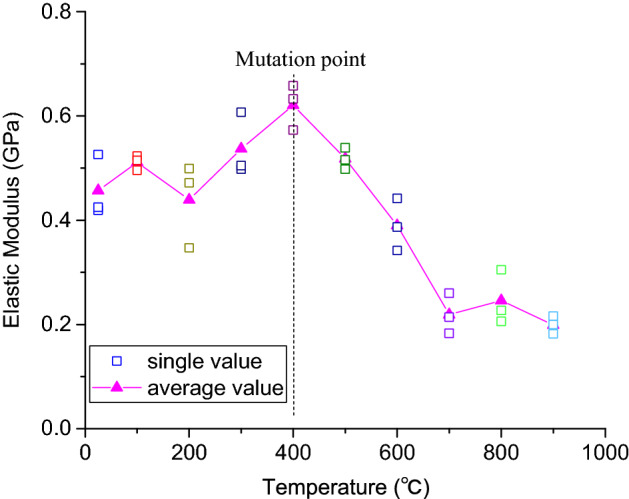
Relationship between the elastic modulus and the treatment temperature of coarse sandstone samples.

The elastic modulus was the most important and characteristic mechanical property of coarse sandstone. It was the characterization of the deformation difficulty of the sample, that is, the slope of the straight line in the stress–strain curve. Figure 9 presents the relationship between the elastic modulus and temperature of the coarse sandstone sample.

Figure 9 shows that the elastic modulus of the coarse sandstone fluctuates with increasing temperature. The elastic modulus increases with increasing temperature after the temperature above 400 °C. Compared with the average elastic modulus of the sample at 25 °C at normal temperature, the average elastic modulus of the sample after 100 °C, 200 °C, 300 °C and 400 °C increases by 11.96%, − 15.75%, 21.30% and 18.53%, respectively. When the temperature exceeds 400 ℃, the elastic modulus of the high-temperature coarse sandstone sample decreases rapidly with increasing temperature, and the elastic modulus of the coarse sandstone sample basically tends to be stable after experiencing high temperature from 700 to 900 °C. Compared with the normal temperature of 25 °C, the average elastic modulus of the samples after high temperature treatment at 500 °C, 600 °C, 700 °C, 800 °C and 900 °C decreases by  − 22.68%,  − 27.86%,  − 37.49%, 5.91% and  − 10.21%, respectively.

### Mechanical strength characteristics

**Figure 10:**
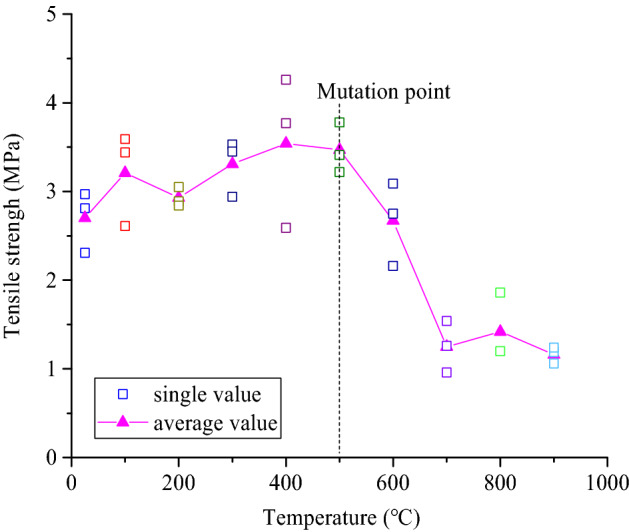
The relationship between the tensile strength of the samples and the treatment temperature.

The relationship between the tensile strength of the coarse sandstone samples treated at different temperatures and the treatment temperature is shown in Fig. 10. The tensile strength of the samples #2and #3 treated at 100 ℃ was higher, and the tensile strength of sample #1 treated at 600 ℃ was lower than that of the other samples at the same treatment temperature. The tensile strength of the samples treated below 500 ℃ increases with increasing temperature. Compared with the average tensile strength of the samples at 25 °C, that of the samples treated at 100 °C, 200 °C, 300 °C, 400 °C, and 500 °C increases by 19.2% and 8.5%, 22.6%, 31.3%, and 28.7%, respectively.

The tensile strength of the samples treated over 500 ℃ decreases with increasing treatment temperature. Compared with the samples at 25 ℃, the average tensile strength of the samples treated at 600 ℃, 700 ℃, 800 ℃, and 900 ℃ decreases by -1.1%, − 53.5%, − 47.3%, and − 56.9%, respectively. This indicates that the treatment temperature below 500 ℃ has a strengthening effect on the tensile strength of the samples; the treatment temperature above 500 ℃ has a weakening effect on that of the samples.

### Mineral component

The main mineral components of the coarse sandstone samples treated at different temperatures are shown in Table 2. The main mineral components of the samples treated at 25 ~ 700 °C were quartz and anorthite. The contents at 25 °C were 62% and 38%; at 100 °C, 67% and 33%; at 200 °C, 63% and 37%; at 300 °C, 66% and 34%; at 400 °C, 71% and 29%; at 500 °C, 69% and 31%; at 600 °C, 48% and 52%; and at 700 °C, 37% and 63.8%, respectively. The inner minerals of the coarse sandstone treated at 800 ℃ undergo a polycrystalline transformation, which results in anorthite disappearance and soda anorthite generation. At this time, the quartz and soda anorthite content was 31% and 69%, respectively. The quartz and sodalite contents of the samples treated at 900 °C were 23% and 77%, respectively. Quartz was the main mineral component of coarse sandstone, and its variation characteristics were consistent with the tensile strength variation trend of the samples.

**Table 2:** Main mineral composition of coarse sandstone after high temperature treament.

Temperature/°C	Chemical reaction	Composition/(%)	Temperature/°C	Chemical reaction	Composition/(%)
25	SiO_2_	62	500	SiO_2_	69
	(Na,Ca)Al(Si,Al)_3_O_8_	38		(Na,Ca)Al(Si,Al)_3_O_8_	31
100	SiO_2_	67	600	SiO_2_	48
	(Na,Ca)Al(Si,Al)_3_O_8_	33		(Na,Ca)Al(Si,Al)_3_O_8_	52
200	SiO_2_	63	700	SiO_2_	37
	(Na,Ca)Al(Si,Al)_3_O_8_	37		(Na,Ca)Al(Si,Al)_3_O_8_	63
300	SiO_2_	66	800	SiO_2_	31
	(Na,Ca)Al(Si,Al)_3_O_8_	34		(Na_0.84_Ca_0.16_)Al_1.16_Si_2.84_O_8_	69
400	SiO_2_	71	900	SiO_2_	23
	(Na,Ca)Al(Si,Al)_3_O_8_	29		(Na_0.84_Ca_0.16_)Al_1.16_Si_2.84_O_8_	77

### Energy characteristic

During the loading process, the loaded coarse sandstone samples continuously accumulate energy. The cumulative energy value was determined by the area enclosed by the deformation curve and the abscissa of the coordinate axis.

5 $$W_{i} = \int_{0}^{u} {p_{i} du_{i} }$$

In Eq. (5), *P* is the load, and *u* is the radial load compression deformation (mm). The integral in Eq. (3) was calculated according to the concept of a definite integral and using the load-deformation curve of the measured samples:

6 $$W_{i} = \frac{1}{2}(p_{i + 1} + p_{i} )(u_{i + 1} - u_{i} )$$

where *n* is the sampling number of the axial load-deformation curve during the test, *i* is the sampling point, and *p* and *u* were the corresponding load and deformation at the sampling point, respectively. Due to the size difference of the samples, the energy accumulated per unit (called the energy rate *W*) was calculated as shown in Eq. 7.

7 $$W = \frac{1}{DL}\sum\limits_{i = 1}^{n} {W_{i} }$$

In Eq. (7), *D* and *L* were the diameter and height of the samples, respectively. The energy analysis in this manuscript refers to the energy per unit area (energy rate) (J/m^2^).

**Figure 11:**
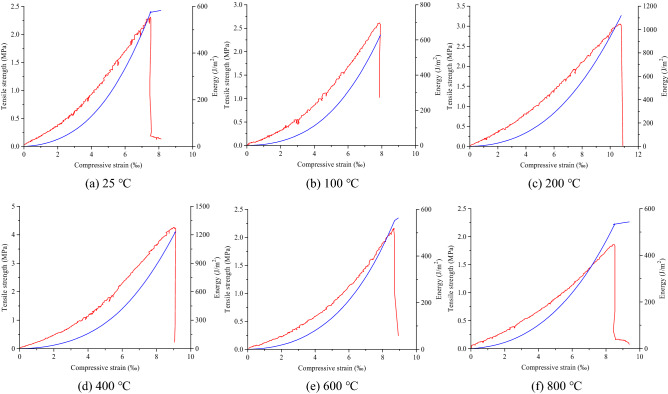
Tensile strength–temperature–energy relationship of the #1 coarse sandstone samples treated at different temperatures.

Figure 11 shows the tensile strength–temperature–energy relationships of the #1 coarse sandstone samples treated at 25 ℃, 100 ℃, 200 ℃, 400 ℃, 600 ℃, and 800 ℃. The tensile strength–temperature–energy relationship of the coarse sandstone samples treated at different temperatures was roughly the same. As the load increases, the compressive deformation of the samples increases gradually, and the cumulative energy also increases accordingly. When the load reaches the peak strength, the energy rate also reaches the maximum value. The sample rupture was accompanied by energy consumption, and then the load decreases rapidly. Compared with the samples at 25 ℃, the cumulative energy of the coarse sandstone samples treated at different temperatures increases by 33.14%, 16.45%, 26.81%, 27.66%, 45.34%, 18.16%, − 55.70%, − 45.39%, and − 57.30%, respectively.

**Figure 12:**
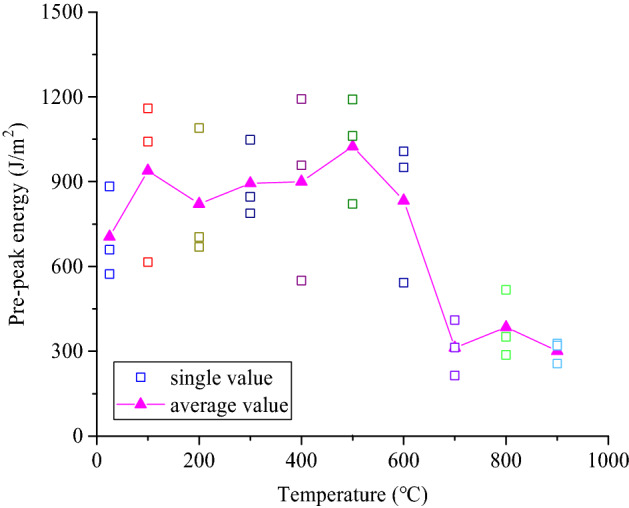
Relationship between the peak energy of the samples and the treatment temperature.

The relationship between the cumulative peak energy and the treatment temperature of the coarse sandstone samples treated at different temperatures is shown in Fig. 12. With increasing treatment temperature, the prepeak cumulative energy of the samples first increases and then decreases, reaching a maximum value at 500 °C. The higher the cumulative energy was, the more rupture surfaces were needed to release the energy. Therefore, a large number of macroscopic cracks were generated in the samples. The energy consumption index was consistent with the macroscopic damage characteristics.

## Discussion

The tensile strength and longitudinal wave velocity of the coarse sandstone samples treated at different temperatures have different degrees of change and have a certain degree of correlation. The relationship between the tensile strength and the longitudinal wave velocity was expressed as shown in Eq. (8):

8 $$\sigma_{t} = x \cdot V_{p}^{y}$$

where *σ*_t_ is the tensile strength (MPa), *V*_p_ is the longitudinal wave velocity (m/s), x and y are constants.

**Figure 13:**
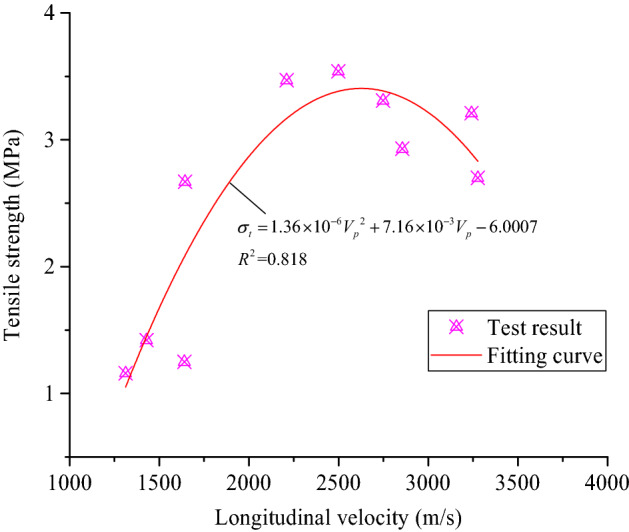
The relationship between the tensile strength and the longitudinal wave velocity.

The coarse sandstone samples was damaged after heat treatment, which reduces the longitudinal wave velocity and changes their tensile strength. The relationship between the tensile strength of the coarse sandstone samples and their longitudinal wave velocity is shown in Fig. 13.

The tensile strength of the coarse sandstone samples treated at different temperatures and their longitudinal wave velocity satisfies a quadratic function fitting relationship, as shown in Eq. (9):

9 $$\sigma_{t} = 1.36 \times 10^{ - 6} V_{p}^{2} + 7.16 \times 10^{ - 3} V_{p} - 6.0007$$

where *σ*_t_ is the tensile strength (MPa), *V*_p_ is the longitudinal wave velocity (m/s).

**Figure 14:**
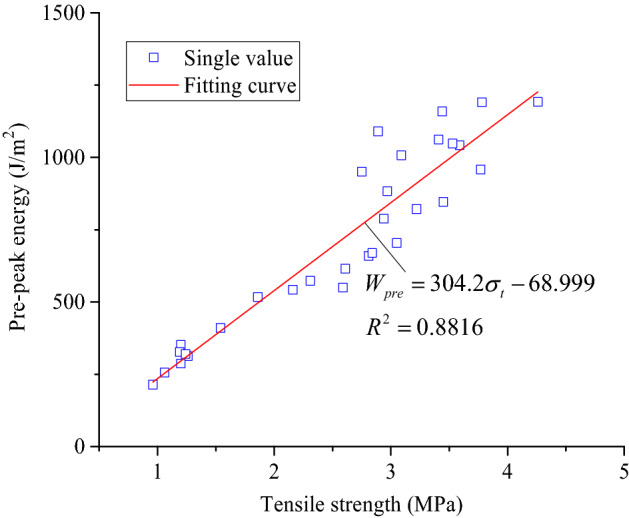
Relationship between prepeak energy and tensile strength of the coarse sandstone samples.

Energy was continuously accumulated inside the coarse sandstone samples during the loading process. When the energy accumulates to a certain level, it was released suddenly, which will leads to instability and failure of the samples, and their stress drops rapidly. The relationship between the prepeak cumulative energy of the samples and their tensile strength is shown in Fig. 14.

The prepeak energy of the coarse sandstone samples and their tensile strength satisfy a linear function fitting relationship, as shown in Eq. 10:

10 $$W_{pre} = 304.2\sigma_{t} - 68.999$$

where *W*_pre_ is the prepeak cumulative energy (J/m^2^),*σ*_t_ is the tensile strength (MPa).

## Conclusion

In this paper, Brazil splitting experiments at different temperatures (room temperature 25 °C and high temperatures of 100 ~ 900 °C) were carried out on the coarse sandstone from a coal seam roof crossed by the Shihuoshan tunnel in Xinjiang, China. The physical and mechanical characteristics of the samples thus were studied, and the following conclusions were drawn:

After heat treatment, the color of the coarse sandstone samples gradually changes from dark gray to brownish red–pink; the higher the treatment temperature is, the darker the sample color. Microcracks and mineral composition changes occur in the coarse sandstone samples after heat treatment, which decreases the longitudinal wave velocity of the samples. The longitudinal wave velocity of the coarse sandstone samples decreases as a quadratic function of the treatment temperature. With the increase in the longitudinal wave velocity, the tensile strength of the samples first increases and then decreases, changing as a quadratic function relationship.

After heat treatment, the tensile stress‒strain curve of the coarse sandstone was divided into compaction, elasticity, plasticity, and failure stages. The energy was continuously accumulated in coarse sandstone before its failure, and it was released suddenly after the failure. With the increase in treatment temperature, the cumulative energy in the prepeak stages first increases and then decreases, reaching a maximum value at 500 °C. The prepeak energy and the tensile strength of the coarse sandstone samples satisfy a linear function fitting relationship.

## Data Availability

Correspondence and requests for materials should be addressed to Y.Y.
